# Rare case of pediatric cecal volvulus

**DOI:** 10.4103/0971-9261.57703

**Published:** 2009

**Authors:** Khizer Mansoor, Sa'ad Al Hamidi, Abdul Mannan Khan, Ram Samujh

**Affiliations:** Department of Pediatric Surgery, Riyadh Medical Complex, Riyadh, KSA; 1 Department of Pediatric Radiology, Riyadh Medical Complex, Riyadh, KSA; 2 Department of Pediatric Surgery, Postgraduate Institute of Medical Education & Research, Chandigarh, India

**Keywords:** Cecal volvulus, intestinal obstruction, malrotation

## Abstract

An 11-year-old female child presented with abdominal pain, vomiting and constipation. An exploratory laparotomy revealed a cecal volvulus due to a congenital band and malfixation of the cecum. This was treated by excision of the band, derotation and decompression of cecum though an appendiceal stump suction.

## INTRODUCTION

Cecal volvulus, one of the manifestations of intestinal malrotation and malfixation anomalies, is very uncommon in children.[1] Limited literature is available on this subject and therefore diagnostic delay can occur. There is no definite consensus on treatment. Conservative management, untwisting with cecopexy, cecostomy and resection with anastomosis have been suggested, although the best long term results have been with resection and anastomosis.[2,3]

## CASE REPORT

An 11-year- old female presented with five days history of mild abdominal pain and two days history of vomiting and constipation. The patient also gave a history of absolute constipation for two days. After conservative management both her pain and distension had settled considerably. There was no history of preceding constipation in the past and no history of a similar episode.

On examination, she was a well developed female child lying comfortably in bed without any obvious pain or distress. She had normal vital signs except mild tachycardia and was mildly dehydrated. Abdomen was hugely distended but moving well with respiration. There was mild generalized tenderness but no point of acute tenderness or guarding. No masses were palpable and bowel sounds were inaudible. Rectal examination revealed an empty rectum.

**Figure 1:**
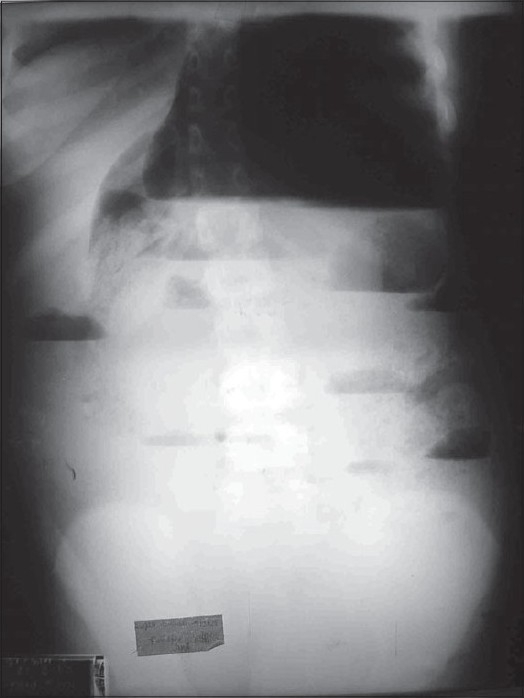
Plain X-ray Abdomen Showing Hugely Dilated Shadow

**Figure 2:**
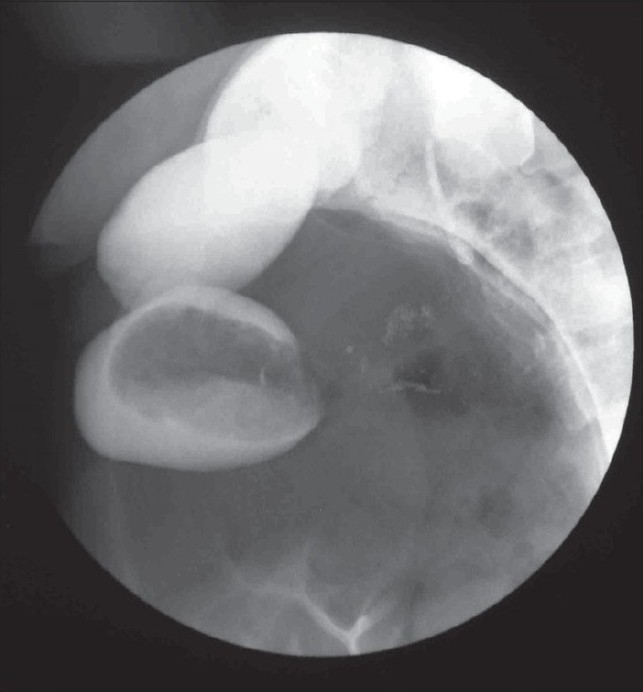
Contrast Enema Showing Failure to Delineate Cecum

Hemogram and serum biochemistry were within normal limits. Plain x-ray abdomen revealed multiple air-fluid levels with a huge gas filled shadow occupying almost whole of left abdomen [Figure 1]. An upper GI contrast study revealed a slow but progressive flow of contrast which could not fill this loop and failed to progress further. A contrast enema delineated the whole of colon except cecum [Figure 2].

**Figure 3:**
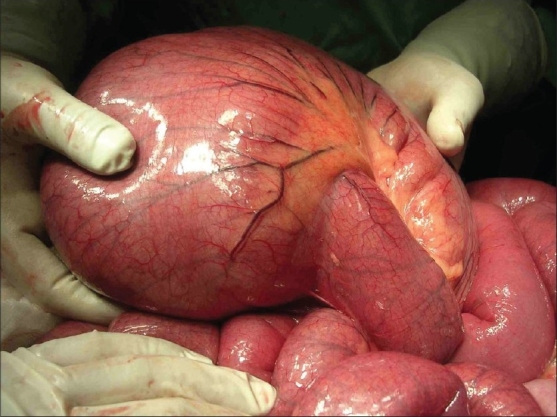
Hugely Dilated Cecum with 270 Degree Volvulus

A provisional diagnosis of obstruction at the level of cecum was made. At laparotomy, a 270 degree counter-clockwise volvulus of the cecum, massively distended, was seen occupying almost whole of the abdomen [Figure 3]. There was a band at the base of this volvulus extending from the posterior peritoneum up to the base of appendix and which probably lead to the acute twisting. There was, however, no vascular compromise. The band was excised and cecum derotated. The base of mesentry was widened. As there was no vascular compromise and the wall of the cecum was appearing acceptable, it was decided that a derotation and emptying of the contents would be adequate. An appendicectomy was done and the stump was used to suck most of the contents. All in all approximately four liters of contents were sucked. The cecum was returned to normal position and no fixations were done.

The patient had an uneventful course except a slightly prolonged ileus. She was discharged home on ninth post-operative day on normal diet with regular bowel movements. She was followed regularly without any complaints and a barium enema done three months later showed almost a normal cecal caliber.

## DISCUSSION

There are many anomalies of intestinal rotation and fixation. They include malrotation in an asymptomatic patient, acute midgut volvulus, chronic midgut volvulus, acute duodenal obstruction secondary to bands, chronic duodenal obstruction, reverse rotation with colonic obstruction behind superior mesenteric vessels, internal hernias (right and left mesocolic hernias) and cecal volvulus.[1,4–6]

Cecal volvulus is mostly seen in adults of over 60 years of age and rarely reported in the pediatric age group.[7] In a series of seven cases of large bowel volvulus in children, two patients had cecal volvulus. Plain abdominal radiographs and barium enemas were diagnostic in these cases.[8] In a large series of 28 cases of intestinal volvulus in general only one case of cecal volvulus has been reported.[9] Common causes in this series were idiopathic, bands or malrotation. It has been reported as a rare association with the Cornelia de Lange syndrome.[10] Cecal volvulus has also been reported as a complication after antegrade colonic enema procedure,[11] after a laparoscopic procedure.[12] or after fundoplication.[13] Although not very frequently, this is regularly reported in adults and detorsion with cecopexy or resection and an end-to-end anastomosis are the suggested treatment options.[14] The rarity of the condition frequently leads to diagnostic delays and complications.[15] Regarding diagnostics, a plain radiograph is suggestive of a haustrated and disproportionately dilated viscus anywhere in the abdomen. Identification of an air-filled appendix attached to this viscus provides evidence of this condition. Barium enema is diagnostic pre-operatively.[16]

In the child under report, symptoms were mild and no evidence of ischemia or peritonitis was observed. Therefore it was considered prudent to go ahead with a contrast study. In a comprehensive review of 48 adult patients different treatment modalities including conservative management, untwisting alone, untwisting with cecopexy, cecostomy and resection with anastomosis have been suggested. However, the best long term results have been with resection and primary anastomosis.[2] Laparoscopic cecopexy has been described more recently.[3]

## References

[1] Robert JT, Edwin IS, James AN, Marc IR, Jay LG, Eric WC, Arnold CG (1998). Pediatric Surgery. Pediatric Surgery.

[2] Ostergaard E, Halvorsen JF (1990). Volvulus of the cecum. An evaluation of the various surgical procedures. Acta Chir Scand.

[3] Madiba TE, Thomson SR (2002). The management of cecal volvulus. Dis Colon Rectum.

[4] Dott NM (1923). Anomalies of intestinal rotation: their embryology and surgical aspects with report of five cases. Br J Surg.

[5] Ladd WE, Gross RE (1941). Abdominal surgery of infancy and childhood.

[6] Gross RE (1953). The surgery of infancy and childhood.

[7] Housni B, Khatouf M, Chater L, Bouabdellah Y, Elomari N, Harandou M (2005). Cecal volvulus: a case report in a child. Arch Pediatr.

[8] Samuel M, Boddy SA, Nicholls E, Capps S (2000). Large bowel volvulus in childhood. Aust NZ J Surg.

[9] Ameh EA, Nmadu PT (2006). Intestinal volvulus: aetiology, morbidity and mortality in Nigerian Children. Pediatr Surg Int.

[10] Holthusen J, Rottingen JA (1998). Cecal volvulus as a complication in Cornelia de Lange syndrome A Case report and literature review. Tidsskr Nor Laegeforen.

[11] Kokoska ER, Herndon CD, Carney DE, Lerner M, Grosfeld JL, Rink RC, West KW (2004). Cecal volvulus: A report of two cases occurring after the antegrade colonic enema procedure. J Pediatr Surg.

[12] Baldarelli M, De Sanctis A, Sarnari J, Nisi M, Rimini M, Guerrieri M (2007). Laparoscopic cecopexy for cecal volvulus after laparoscopy. Case report and review of the literature. Minerva Chir.

[13] Simpson ET, Keating S (1991). Price J Cecal volvulus in a child: An unusual postoperative complication. Aust NZ J Surg.

[14] Gupta S, Gupta SK (1993). Acute cecal volvulus: report of 22 cases and review of literature. Ital J Gastroenterol.

[15] Consorti ET, Liu TH (2005). Diagnosis and treatment of cecal volvulus. Postgrad Med J.

[16] Young WS (1980). Further radiological observations in cecal volvulus. Clin Radiol.

